# Did the first Global Fund grant (2003–2006) contribute to malaria control and health system strengthening in Timor-Leste?

**DOI:** 10.1186/1475-2875-11-237

**Published:** 2012-07-23

**Authors:** João Soares Martins, Anthony B Zwi, Paul M Kelly

**Affiliations:** 1 Faculty of Medicine and Health Sciences, Universidade Nacional Timor Lorosa’e, Dili, Timor-Leste; 2 Health, Rights and Development (HEARD@UNSW), School of Social Sciences and International Studies, Faculty of Arts and Social Sciences, and formerly GlobalHealth@UNSW and the School of Public Health and Community Medicine, University of New South Wales, Sydney, Australia; 3 Medical SchoolAustralian National University, Canberra, ACT, Australia; 4 Population Health Division, ACT Government Health Directorate, Canberra, ACT, Australia

**Keywords:** Global Fund to fight HIV/AIDS, Tuberculosis and malaria, Malaria control, Capacity building, Health system, Additionality, Timor-Leste

## Abstract

**Background:**

In 2003, Timor-Leste successfully obtained its first Global Fund grant for a three-year programme for malaria control. The grant aimed to reduce malaria-related morbidity and mortality by 30 % by the end of the implementation.

**Methods:**

A mixed-methods approach was used to assess the impact of the grant implementation. Fifty-eight in-depth interviews, eight group interviews, 16 focus group discussions, and on-site observations were used. Morbidity data reported to the Ministry of Health were also examined to assess trends.

**Results:**

The National Malaria Programme with funding support from the Global Fund grant and other development partners contributed considerably to strengthening malaria control and the general health system. It also brought direct and indirect benefits to pregnant women and to the community at large. However, it failed to achieve the stated objective of reducing malaria morbidity and mortality by 30 %. The implementation was hampered by inadequate human resources, the rigidity of Global Fund rules, weak project management and coordination, and inadequate support from external stakeholders.

**Conclusion:**

Despite limitations, the grant was implemented until the agreed closing date. Considerable contributions to malaria control, health system, and the community have been made and the malaria programme was sustained.

## Background

The Global Fund to Fight AIDS, Tuberculosis and Malaria (referred to hereafter as the Global Fund) was created in January 2002 with the aim of mobilizing resources from governments, donors, the private sector, and individuals to tackle HIV/AIDS, tuberculosis (TB) and malaria [1,2]. At that time, these three diseases were estimated to kill more than six million people each year [3].

The Global Fund presents itself as a “performance-based funding” mechanism which requires proposals to be submitted by recipient countries. These proposals need to be developmentally sound and technically implementable. This process aims to ensure that grant investments are managed and spent effectively on programmes in the fight against the three target diseases [4]**.** The Global Fund operated as a financial instrument providing funds to support country-led disease control programmes, not as an implementing entity [5].

A three-year Global Fund grant for malaria control in Timor-Leste was signed by the Ministry of Health and the Global Fund in June 2003. This first Global Fund grant, valued at USD 2,876,903, had an overall objective of reducing malaria morbidity and mortality by 30 % by the end of the project [6].

As is standard practice for Global Fund grants, and to facilitate the implementation, the County Coordinating Mechanism (CCM) was formed, and the Principal Recipient (PR) and Sub Recipients (SRs) identified. The Local Funding Agent (LFA) was appointed with the responsibility to supervise and recommend funding disbursement for the programme implementation. The Ministry of Health (MoH) was appointed as PR and the Minister for Health took on the role of CCM chair. A project management unit (PMU) was established to manage the implementation of the grant.

Timor-Leste has been awarded five Global Fund grants: the malaria grant in 2003; a TB grant in 2005 which was suspended in 2006; an HIV/AIDS grant in 2006; and second grants for TB and malaria in 2009. The focus of this study is on the first Global Fund grant for national malaria programme from 2003 to 2006.

This study describes the results produced through the Global Fund supported programme on malaria control, analyses whether Timor-Leste abided by the Global Fund’s *principle of additionality*, and examines the impact of the Global Fund on national malaria control and the health system, as well as the challenges faced during grant implementation.

It is worthwhile to note some terminologies are used interchangeably in this study to refer to the national malaria control programme with funding support from the Global Fund. These terms are “malaria control program funded by the Global Fund”, “malaria control program supported by the Global Fund”, “malaria control program with funding support from the Global Fund”, “the Global Fund funded malaria program”, and “the Global Fund grant”.

## Methods

The study used both qualitative and quantitative methods. The qualitative methods comprised in-depth interviews, focus group discussions (FGDs), observation, and site visits. The quantitative methods used routinely collected malaria morbidity data reported to the MoH from 2004 to 2008.

The study purposefully assessed the MoH in its capacity as the PR of the Global Fund grant, the PMU, CCM, SRs, World Health Organization, donors, District Health Services, local authorities and community, and NGOs who implemented the malaria programme but were not funded by the Global Fund. Six SRs were selected for study on the basis of size of the grants received and the type of interventions they were contracted to undertake. They were the Communicable Disease Control (CDC) Department of the MoH, HealthNet International (HNI), World Vision International (WVI), the Christian Children’s Fund (CCF), Timor Aid, and Resatil (a local NGO). Seven of 13 districts (Aileu, Baucau, Bobonaro, Covalima, Dili, Manufahi, and Viqueque) in which the SRs were active, were purposively selected for the study (Table 1).

**Table 1:** Characteristics of key informants, group interviews and focus group participants

**Institutions**	**Key informant interview**	**Group Interview**	**Focus Group Discussion**
National Level			
*MoH*	10	0	0
*PMU and CCM*	6	0	1
*WHO*	3	0	0
*SRs*	10	0	0
*TAIS*	1	0	0
*CARE International*	1	0	0
Districts and CHCs	8	5	6
Health cadres and teachers	5	1	0
Local Authority	5	0	0
Community members	0	0	9
SR field officers	6	2	0
Donors	3		
**Total**	**58**	**8**	**16**

In addition, in some of these selected districts, some malaria programmes were implemented by different organizations, which were not funded by the Global Fund. In Baucau, Viqueque, and Aileu, an ITN distribution programme was implemented by Timor-Leste Assistensia Saude Integradu (TAIS), a USAID-funded agency which supports the MoH, while a CARE International malaria programme was also implemented in Bobonaro and Covalima.

The document review was undertaken to the following documents: the country proposal submitted to the Global Fund, work plans, quarterly reports, annual reports, LFA reports, minutes of meetings of the CCM, and official correspondence related to the Global Fund operation in Timor-Leste. Documents from TAIS and CARE were also collected and reviewed.

In total, 58 open-ended interviews, eight group interviews, and 16 FGDs were conducted. Twelve visits to project sites (eight *aldeias* [villages] and four primary schools) were undertaken to assess the impact of the programme on local communities. An in-depth interview was an interview conducted between interviewer and interviewee (a key informant). There is a slight difference between group interviews and focus group discussions [7-9]. A group interview referred to an in-depth interview with a group of people aiming at exploring ideas with communication being bi-directional between the interviewer and the interviewees without involving discussion among participants. Focus group discussion is a multi-directional communication which permitted participants in the group to discuss issues among themselves and also with the interviewer.

The key themes addressed in this study were:

What were the results seen in malaria control programme and to what extent the Global Fund grant contributed to these results.

Whether or not Timor-Leste abided by the Global Fund’s principle of additionality

To what extent the Global Fund impacted on national malaria control and the health system

What were the views of beneficiaries and the community on malaria control

What were the challenges faced during grant implementation.

This assessment is also looking at how the malaria control programme supported by the Global Fund contributed to health system strengthening. “Health system” is understood as the sum total of all the organizations, institutions, and resources whose primary purpose is to promote, restore, and maintain health [10]. Since the malaria control programme is also part of the health system, therefore, in this particular setting, this study also intends to assess how the Global Fund grant contributed to improving malaria control and the wider health system in Timor-Leste’s context.

The quantitative data analysis was directed at analysing the annual malaria incidence (AMI) rates from 2004 to 2008. This was calculated using the total annual malaria cases from 2004 to 2008 divided by the total population and multiplied by 1,000. The total yearly malaria cases of 2004 to 2008 was obtained from the Ministry of Health and the data on population numbers (924,624) were obtained from the 2004’s Census Report [11], with a projection of annual population growth of 3.13 % [12] to take account of the increase of population numbers in the subsequent years after 2004.

Since the objective of the Global Fund funded malaria programme was to reduce malaria morbidity and mortality by 30 % at the end of the project, the 2004’s AMI was used as the baseline to assess whether the Global Fund programme had contributed to the reduction of malaria morbidity. Negative binomial regression was used to estimate the incidence rate reduction for each year relative to 2004. The number of malaria cases was the dependent variable with year (categorical) as the independent variable and the natural logarithm of population size as the offset variable. This was done using Stata (Release 11.2)*.*

Information derived from in-depth interviews, group interviews, and FGDs were transcribed, translated into English, and coded with NVivo 7. Both content and thematic analyses were conducted and these were cross-checked with data from document reviews. Triangulation of qualitative and quantitative findings was undertaken.

Ethical clearance was obtained from the Ethics Committee of the University of New South Wales. In addition, a permission letter from the Ministry of Health Timor-Leste was also sought. Prior to interviewing participants, informed consent was obtained from them in writing and verbally for any participants who were illiterate.

## Results

The results’ presentation begins with a description of the outcomes of the intervention, and analysis of the impacts, additionality, and challenges.

### The outcome of the Global Fund intervention

The intervention areas and the key outcomes of the Global Fund interventions are presented in Table 2.

**Table 2:** Summary results of the Global Fund intervention in Timor-Leste

**Sub-Recipient**	**Intervention areas**	**Results**
MoH	Improved EDPT	Conducting small scale of training and pilot testing of RDT test, and microscope training
	Health System strengthening	Establishing a malaria unit and recruiting national malaria staff, international experts, establishing IDSS, producing of an epidemiology bulletin. Training more than 268 health staff on a malaria treatment protocols and 472 on surveillance. Producing a manual for IDSS
	Behavioural communication change (BCC) programme	Producing leaflets, posters and brochures
HNI	Care, Prevention and Control	Establishing the ITN distribution system via integration with antenatal care services throughout the country and distribution of 68,228 ITN to pregnant women and training 686 health staff on ITN, and research.
WVI	BCC programme	Programme reached 52,779 people, village health clean up, one off ITN distribution to people in one of the sub-district understudy.
CCF	BCC Programme	Establishing 47 local health committees, training of 141 traditional healers, 50 health volunteers trained and more than 7,000 attended health education programme
Timor Aid	BCC Programme	Collaborating with MoH and DHS and CHCs, 805 health volunteers trained and 36,419 people attended health education programme
Resatil	Small Grant Scheme	Collaboration with the Ministry of Education and training 3,292 student and 127 teachers
Unpaz*	KAP Study	Conducting KAP in 4 districts (Baucau, Dili, Lautem, and Manufahi)
Laboratory Cito*	Small Grant Scheme	Training on microscopic examination in 5 CHCs in Lautem district
Vinset and HHU*	Small Grant Scheme	Implementing School Health Programmein 47 primary schools in Dili and Manatuto districts and reached 5,388 students and 356 teachers

* = Sub Recipients not included in the sample.

### Malaria morbidity trends

**Figure 1:**
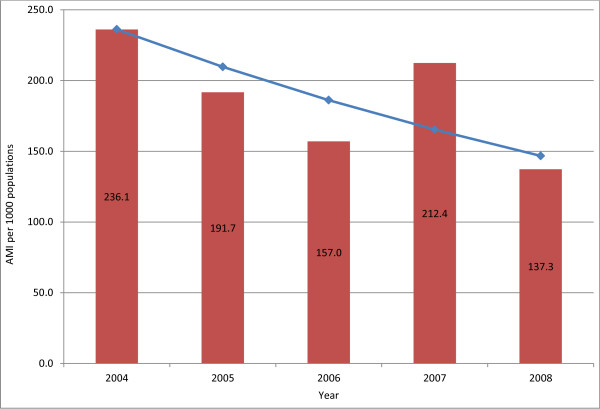
The AMI per 1,000 populations in Timor-Leste 2004–2008.

The AMI rate for 2004 was 236.1 per 1,000 populations and this started to decrease in 2005 and 2006, but increased again in 2007 to 212.4 per 1,000 populations (Figure 1). In 2008, the AMI declined again to 137.3 per 1,000 populations. Since 2004’s AMI was considered as the baseline and 2007’s AMI as the end point, an overall reduction was around 10.1 % (95%CI 9.6 – 11) P < 0.001, which is still far from the stated goal of 30 % morbidity reduction. However, when using the 2008 figure, the reduction from 2004’s baseline was 41.9 % (95%CI 41.5 – 42.3) P < 0.001. Therefore, the reduction of AMI was statistically significant.

### Global Fund contributions to national malaria control and the health system

In total, there were 34 sources of comments (30 key informants, two FGDs, and two group interviews) on whether the Global Fund intervention in Timor-Leste made any contribution to capacity building, malaria control, the health system, knowledge change, or the community.

The contributions to malaria control included the establishment of a malaria unit, recruitment of malaria unit officers, development of the National Malaria Strategy, training of health staff on treatment protocols, assignment of international malaria and entomology experts, implementation of insecticide treated-mosquito nets (ITNs) distribution, and health promotion activities at community level.

"“Global Fund’s contribution is the establishment of malaria unit, initiation of entomological studies and interventions, and establishing some control measures” (A UN Agency Country Representative 2001–2007)."

A contribution to health system strengthening was evident through the establishment of an integrated disease surveillance system (the IDSS), the implementation of rapid diagnostic testing (RDT), and the setting up of vector control management. Improved staff knowledge and experience also supported health system strengthening. Health system strengthening and capacity building are interlinked, thus investment in either ultimately provided benefits to both the system and individuals in the system. It was acknowledged that the Global Fund improved staff knowledge in managing malaria both clinically and programmatically.

"“Regarding capacity building it is for example training, malaria protocol, we developed our protocol, train people how to do things according to the protocol, the funding was from them [the Global Fund]…it increased also the capacity of colleagues in the area of treatment, [health] promotions and education for malaria” (A senior officer at MoH)."

Benefits for capacity building were personally felt by those involved in the Global Fund implementation.

"“For me, I learned many things, how to implement programmes, and with whom to coordinate. And with this [Global Fund] programme, it increased my experience, how to deal with community and how to deal with local authority, how to prepare people…I learned how to become a good facilitator… and to respond to what the community wants” (NGO/SR Field Officer)."

### Global Fund’s benefits for community

Despite the challenges and constraints faced during the implementation phase, the support funding from the Global Fund contributed to health promotion activities and ITN distribution. The Ministry of Health’s Reports indicated that nearly 10 % of the country’s population (93,444 reported data) were reached through health education and promotion activities [13-15]. Between 2004 and 2006, pregnant women and, in some instances, the general population had access to free ITNs.

"“For pregnant women, there is a positive contribution. Because they get mosquito net, we hardly see malaria in pregnant women, and malaria with complication…Information reached the community. Pregnant women passed information to other pregnant women, some demanded us to give them nets” (FGD with CHC staff)."

The findings from the Timor-Leste’s Survey of Living Standard (TLSLS) in 2007 found 51.8 % of the population, of all ages, slept under mosquito nets (including 18.8 % using ITNs), and 62.9 % of children under the age of five (including 21.8 % using ITNs). While the survey from 2001 showed that 41.3 % of the total population slept under mosquito nets as did 51.8 % of children under age of five [16], the above figures suggest there had been an increase of over 10 % in overall mosquito net utilization in 2007 as compared to 2001. The TLSLS’ findings highlighted the benefit of the malaria programme supported by the Global Fund on the community.

The Global Fund’s interventions also contributed to knowledge change about malaria.

"“The positive contribution, in the community’s attitude we see change, I see this, because they have some understanding. They know it, by knowing this, their attitude also changes, and also severe malaria has also come down, because when they have symptoms, they have awareness to come, they are not waiting, this is positive” (NGO senior officer)."

The positive change was not only reported by programme implementers, one community health volunteer made a remark on changes in attitudes and behaviour after being involved in a training programme:

"“Even if we have stopped now… we know a bit about malaria because this is a malaria area, the training was exactly about malaria, when we come across people like this we always refer them to hospital, even if they said they believe their traditional medicine, we advise them to go to hospital” (Group Interview with Health volunteers in Alas, Manufahi)."

Community members also noted the positive results derived from the malaria interventions ranging from free ITN distribution, the reduction of malaria cases in their community, provisions of information of malaria, and awareness of cleaning their environment.

"“Success, the mosquito net distribution can be regarded as a success…people know more about malaria. Pregnant women get mosquito nets to protect them. We know mosquitoes live in dirty environment. Malaria information reached the community. People started to clean their environment” (FGD with community in Bobonaro)."

"“We got mosquito nets free… Malaria cases appeared to be going down” (FGD with community in Covalima)."

Communities also expected NGOs to conduct training and refresher courses continuously, and also demanded that their fellow volunteers deliver health messages to the community “*to continue keeping us warm with information”*.

### Did the additionality principle apply in Timor-Leste?

One of the seven Global Fund’s principles is additionality. This means that “the Global Fund makes available and leverages additional financial resources” [5]. Therefore, the Global Fund grants should not be used to substitute funds provided by other sources.

Overall, the Global Fund contributed around 8 % of the total MoH budget for three fiscal years (Table 3), excluding funds from other donors for the malaria control programme. This was derived from comparing the expenditure of the state budget allocated to the MoH in the fiscal years 2003–2004, 2004–2005, and 2005–2006 with the Global Fund disbursement in the same fiscal periods.

**Table 3:** Government budget for health in comparison with funding from the Global Fund in 3 Fiscal Years (2003-2006) in US Dollars

**Fiscal Year**	**Government Budget for the MoH ($000s) USD**	**Budget from the Global Fund ($000s) USD**	**% funds derived from the Global Fund in comparison with the MoH budget**
2003-2004	8,853	656.1	7.4%
2004-2005	9,806	1,005	10.3%
2005-2006	16,884	1,031.7	6.1%
Total 3 Fiscal years	35,543	2,692.8	7.6%

MoH: Ministry of Health

For a single disease (malaria), it was indeed a considerable budget, however, the MoH considered that the support was still additional support by arguing that the MoH provided support to enable the grant to operate in terms of providing infrastructure, health workers, and drugs:

"“Generally speaking it was additional funding, even though some may argue that the Government of Timor-Leste did not put on the table, for example, an X amount of funding to correspond with the Global Fund […]. If you look carefully the structures of implementation, the mechanisms of implementation, let’s say for example, [the] malaria programme, [the] CDC used all the District Health Services, which received budget from government.” (A most senior MoH official)."

The Global Fund had not displaced or deterred other donors from supporting the malaria programmes. In fact, other implementers outside the Global Fund, such as CARE International and TAIS, also implemented malaria control programmes in some districts. Therefore, this implies that the additionality principle was generally applied in Timor-Leste.

### Challenges in the Global Fund programme implementation

The implementation of the Global Fund supported malaria programme faced a number of challenges as it was a new initiative at the global level.In Timor-Leste, those who were appointed or got involved in the PR, CCM, and SRs at that time had little knowledge about the grant. The PMU had limited capacity in project management and the CCM did not have sufficient resources, which constrained it from performing its oversight role. This was further aggravated by the lack of human resources to support the programme because the Malaria Unit had only two project officers. In addition, there were delays in the recruitment of international advisers for supporting programme management and disease control. All these slowed the pace of implementation, which prompted the Global Fund to issue a warning letter advising that both the malaria grant and the TB grant had been listed in the Global Fund’s Early Alert and Response System (EARS) List. Monitoring and evaluation was very weak and noted several times by the LFA. Late funding disbursement impacted on the implementation, as funding can only be made when all SRs met the minimum quarterly indicators. Conversely, slow implementation led to late funding disbursement.

There were imbalances in funding allocation to the four strategies for controlling malaria. Case management and vector control, arguably the most effective interventions for malaria control, were grossly underfunded. Most of the fund went to behavioural change (36 %), ITN distribution (35 %), health system strengthening (15 %), and project management (13.5 %). Funding for case management (improved diagnosis) was less than 1 %. Funding for vector control through the application of fish farming was proposed but failed to be implemented, thus this funding was re-programmed to other activities. Though re-programming of under-performed programmes was permitted by the Global Fund [17], this indicated the inadequate planning.

The rules of the Global Fund were believed to be too rigid. Substantial bureaucratic constraints in the management of the Global Fund were noted and at times led to frustration.

"“The Global Fund seems to care more about structuring the process than the outcome…we should not be the slave of the process with no result” (A UN Agency Country Representative in Timor-Leste)."

The PMU not only administered the project but also managed its programmatic aspects, which created confusion with the CDC department’s role. The CDC was a SR of the Global Fund grant,which impeded CDC in overseeing programmes implemented by other SRs because they reported directly to the PMU.

"“This is something not clear to me, we [The CDC department] run malaria programmes in the whole territory, whoever wants to do something on malaria programme, they should be in line with the malaria programme. [However] at that time the status of CDC changed to Sub Recipient, so the role of CDC was limited, we did not have access to other Sub Recipients, because we were all Sub Recipients” (Malaria Programme Officer, MoH)."

Lack of coordination resulted from poor communication. Districts were poorly informed about the Global Fund programmes. In some cases, the Global Fund programme had been wrongly perceived as a “NGOs’ programme”. Sometimes the NGOs themselves failed to inform the District Health Services of their activities.

"“The ITN distribution programme belonged to MoH, but DHS [District Health Service]did not know…I told them ‘this is an MoH programme, you need to implement it’… but these colleagues said ‘no this is from NGO, you get money for that, now you talk about this, you try to make the project success, but you lie to us’” (NGO field manager)."

**Figure 2:**
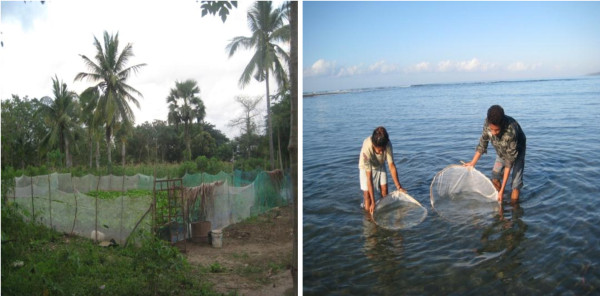
**Misuse of ITNs for other purpose.a**) Protecting vegetables; **b**) catching fish and prawns.

Lack of awareness led to some misuse of ITNs; for example, using nets to cover plants or for fishing. This was observed by one of the researchers (JM) during fieldwork (Figure 2) and has been documented by another study [18].

"“Some without being aware of this and take it [ITN] to catch fish in river and ponds. Some even just hang it on the walls and not using it” (NGO Senior Officer)."

Some communities tended to participate more in programmes that provided goods; for example, ITNs, or being paid, rather than in those that only offered health education.

Donor agencies at country level did not provide support to the implementation of the first Global Fund grant. However, there were arguments that for such support to happen, the government should identify areas where it needed help from donors.

"“There is no reason why we [donor communities] couldn’t provide support [to the Global Fund] at country level but that’s more difficult, it is more difficult in a sense that the governments would have to say that this is a priority” (Senior AusAID Adviser)."

Presently the situation has changed where both multi-lateral and bi-lateral donors have their representation in the CCM body, but this was not the case with the first grant. The grant implementation was interrupted by political instability and civil unrest in 2006, which resulted in a six-month exceptional extension from the original closing date of December 2006 [19].

## Discussion

Timor-Leste lacked infrastructure and human resources when it applied for its first Global Fund grant; the country had just secured its independence in May 2002. Despite all the constraints and limitations present, Timor-Leste managed to successfully implement the three-year Global Fund malaria programme grant.

Globally, it is acknowledged that the Global Fund has improved the coverage and quality of services for HIV/AIDS, TB and malaria control [20]. In Timor-Leste, there was a positive impact on malaria morbidity reduction although this was only around 10 % at the end of the implementation period, below the overall goal of a 30 % reduction. By the following year, however, further reductions were noted. However, it needs to be emphasized that the morbidity reduction was not solely due to the supports of the Global Fund and other development partners, government contributions in terms of supporting the provision of infrastructure and human resources in the wider health sector also contributed to this outcome.

This reduction in morbidity is less than seen in other countries such as Rwanda (61 %) and Ethiopia (75 %) through rapid scale-up of ITN and ACT coverage [21-23]. In Laos, there was a reduction of 12.3 % of confirmed malaria cases from 2005 to 2009 [24].

The imbalance of resource allocation towards ITN distribution and behavioural communication change, and the underfunding for diagnosis, treatment and vector control interventions, and the political instability in 2006, are likely to have contributed to the Global Fund not achieving its objectives. A study conducted earlier to assess the impact of political instability on malaria control suggested that the instability in 2006 contributed to the increase of malaria rates in 2007 [19].

A robust health system is generally seen as a pre-requisite for the success of implementing donor support programmes [25]. In Timor-Leste, the Global Fund grant made a significant contribution to capacity building and to the broader health system, which in the long-run is likely to further facilitate reduction of morbidity and mortality due to malaria, and most likely to other diseases. The utilization of mosquito nets, including ITNs, appeared to have increased by a little more than 10 % (approximately 41 % in 2001 to 52 % in 2007 for the general population, and for children under age of five, the increase was from 52 % in 2011 to 63 % in 2007) [16]. However, this study also noted a few sporadic misuses of ITNs for other purposes (see Figure 2). The misuses of ITNs have been reported by another study [18]. Therefore, continuous efforts need to be made to maintain these gains, as suggested by one informant, “*continue to keep us warm with information*” and beyond that translating this knowledge into practice. The government, implementing partners, and the community should work hard to convince people not to misuse ITNs for purposes other than to protect them from mosquito bites thus protecting them from mosquito-borne diseases including malaria.

Based on the MoH annual reports, the health education programme reached 93,444 people (around 10 % of the country’s population) at that time [13-15]. This was made possible by a notable contribution from SRs of the NGO sector. An earlier study documented that about 90 % of the sampled population knew about malaria terminology but there were also misconceptions about malaria causation and transmission [26]. This study found an improved community understanding of malaria and also documented that communities knew about the malaria programmes being implemented in their villages.

The implementation of the Global Fund funded programme also created partnership with the non-government sector as the implementing partners for the MoH in fighting HIV/AIDS, TB and malaria at country level. This study also revealed that funding from the Global Fund was an additional resource as demonstrated by the commitment of other donors, particularly the European Commission (through Care International) and USAID (through TAIS), who also funded malaria interventions. Therefore the reduction of malaria morbidity, strengthened malaria programme and general health systems should be seen as a result of combined efforts from the Global Fund funded programmes for malaria, contributions from government and donors outside the Global Fund.

The lack of capacity within the Global Fund structures at national level reflected the lack of available expertise within the country and delays in seeking external technical assistance, even though, the Global Fund guidelines permitted the PR to seek technical assistance (TA) to support proposal development and grant implementation [27]. This was a key contributor to both the malaria and TB grants in Timor-Leste being put under the Global Fund’s EARS List. This highlights the importance of basic management and system capacity to ensure effective implementation [28]. To a certain extent, other donor agencies (for example, the World Bank, AusAID and USAID) from countries which had contributed funding to the Global Fund centrally in Geneva (not necessarily at country level), could have offered assistance to support the implementation of the Global Fund grant. The grant could have achieved more if supported with adequate human resources, infrastructure, and general support. Poor coordination among participants involved in the implementation, lack of capacity in project management, and weak monitoring and evaluation contributed to low absorptive capacity and slow progress in grant implementation in Timor-Leste. Poor coordination, weak monitoring and evaluation were major hurdles not only for implementation of this grant, but also in the implementation of a new Malaria Treatment Protocol applied in 2008 [9]. Absorptive capacity had been a major concern raised by donors during the discussions of the Global Fund establishment [29]. For example, Zambia also experienced poor coordination and insufficient monitoring and evaluation affecting its Global Fund grant implementation [30].

The key tenet of the Global Fund is that of “performance-based funding” and is seen as key for achieving measurable results. However, at the same time, many countries including Timor-Leste, have found the system burdensome, rigid, and fixed exclusively on the measurement of process indicators and short-term outputs rather than on key longer-term outcomes, results and capacity building. The quote *“We should not be the slave of the process with no result”* carries important messages for improvement. The programme should strive to achieve its defined objectives and goals with the processes in place supporting the achievement of such longer-term impact.

### Strengths and limitations

The strength of this study is that it documented the results of the Global Fund support programme on Malaria Control in Timor-Leste and its wider impact on the morbidity reduction as well as on health system strengthening. It captured perspectives of actors involved in the grant implementation and views of the beneficiaries and communities on the benefits of the programme. It also revealed factors that impeded the implementation of this grant.

The use of morbidity data reported to the Ministry of Health demonstrated the impact of the Global Fund intervention particularly towards morbidity reduction, to some extent. Many studies have highlighted the importance and validity of routinely collected statistical data on morbidity and mortality [31-34]. However, there are limitations inherent to the use of routinely collected data as it may not fully reflect the actual incidence of malaria, it may result in either overestimate and underestimate the burden of disease burden itself. The use of clinically diagnosis approach in detecting malaria cases prior to the introduction of rapid diagnostic test in 2008 may over-diagnose malaria cases and thus affect the estimate of incidence rates. This can be considered as a potential limitation for this study. Therefore, it is important to exercise caution when interpreting routinely morbidity collected data.

## Conclusions

Despite failing to reach the intended objectives in the expected time frame, the Global Fund grant provided considerable resources to the malaria control programme and has contributed to health system strengthening, capacity building, improved community knowledge, and the provision of free mosquito nets to the community. Reduction of malaria incidence in subsequent years was made possible through a sustained government programme in malaria control and also with funding and technical support from the Global Fund and other development partners. The rigidity of the Global Fund rules, weaknesses in project management, lack of human resources, the political instability in 2006, and lack of support of the wider donor community at country level affected the slow progress in the Global Fund grant implementation.

The important lessons to be learned from the first Global Fund grant are that clearly defined roles of participants involved, necessary infrastructure, human resources, knowledge on grant requirements and conditions, strong project management and coordination are keys to success in implementation. When the expertise needed to facilitate grant implementation is not available within a recipient country, procuring this expertise should occur as early as possible to ensure that the grant meets its objective and brings positive impact to improving malaria control, benefiting the affected population and strengthening the health system more generally.

## Competing interests

The authors declare no competing interests.

## Authors’ contributions

JM completed his PhD at the University of New South Wales. This study was part of his PhD thesis. JM was involved in conceptualizing this study, conducting data collection, data analysis, writing the first draft of this paper and contributed to all stages of the paper until finalization. AZ supervised JM PhD studies. AZ contributed to conceptualizing this research and data analysis, and contributed to each stage of the write up and finalization of the paper. PK co-supervised JM's PhD studies. PK was involved in study design, data analysis and presentation, and all aspects of the write-up for publication. All authors read and approved the final manuscript.
